# Apoptosis Imaging in Oncology by Means of Positron Emission Tomography: A Review

**DOI:** 10.3390/ijms22052753

**Published:** 2021-03-09

**Authors:** Christophe Van de Wiele, Sezgin Ustmert, Bart De Spiegeleer, Pieter-Jan De Jonghe, Mike Sathekge, Maes Alex

**Affiliations:** 1 Department of Nuclear Medicine AZ Groeninge, 8500 Kortrijk, Belgium; sezgin.ustmert@gmail.com (S.U.); PIETERJAN.DEJONGHE@azgroeninge.be (P.-J.D.J.); alex.maes@azgroeninge.be (M.A.); 2 Department of Diagnostic Sciences, University Ghent, 9000 Ghent, Belgium; 3 Department of Analytical Chemistry, DRUQUAR, University Ghent, 9000 Ghent, Belgium; Bart.DeSpiegeleer@UGent.be; 4 Department of Nuclear Medicine, University of Pretoria, Pretoria 0084, South Africa; Mike.Sathekge@up.ac.za; 5 Department of Morphology and Imaging, University Leuven, 3000 Leuven, Belgium

**Keywords:** apoptosis, positron emission tomography, oncology

## Abstract

To date, a wide variety of potential PET-apoptosis imaging radiopharmaceuticals targeting apoptosis-induced cell membrane asymmetry and acidification, as well as caspase 3 activation (substrates and inhibitors) have been developed with the purpose of rapidly assessing the response to treatment in cancer patients. Many of these probes were shown to specifically bind to their apoptotic target in vitro and their uptake to be enhanced in the in vivo-xenografted tumours in mice treated by means of chemotherapy, however, to a significantly variable degree. This may, in part, relate to the tumour model used given the fact that different tumour cell lines bear a different sensitivity to a similar chemotherapeutic agent, to differences in the chemotherapeutic concentration and exposure time, as well as to the different timing of imaging performed post-treatment. The best validated cell membrane acidification and caspase 3 targeting radioligands, respectively ^18^F-ML-10 from the Aposense family and the radiolabelled caspase 3 substrate ^18^F-CP18, have also been injected in healthy individuals and shown to bear favourable dosimetric and safety characteristics. However, in contrast to, for instance, the ^99m^Tc-HYNIC-Annexin V, neither of both tracers was taken up to a significant degree by the bone marrow in the healthy individuals under study. Removal of white and red blood cells from the bone marrow through apoptosis plays a major role in the maintenance of hematopoietic cell homeostasis. The major apoptotic population in normal bone marrow are immature erythroblasts. While an accurate estimate of the number of immature erythroblasts undergoing apoptosis is not feasible due to their unknown clearance rate, their number is likely substantial given the ineffective quote of the erythropoietic process described in healthy subjects. Thus, the clinical value of both ^18^F-ML-10 and ^18^F-CP18 for apoptosis imaging in cancer patients, as suggested by a small number of subsequent clinical phase I/II trials in patients suffering from primary or secondary brain malignancies using ^18^F-ML-10 and in an ongoing trial in patients suffering from cancer of the ovaries using ^18^F-CP18, remains to be proven and warrants further investigation.

## 1. Introduction

Apoptosis, a form of programmed cell death first described by Kerr et al., is a natural, orderly energy-dependent process that causes cells to die without inducing an inflammatory process [1].

**Figure 1:**
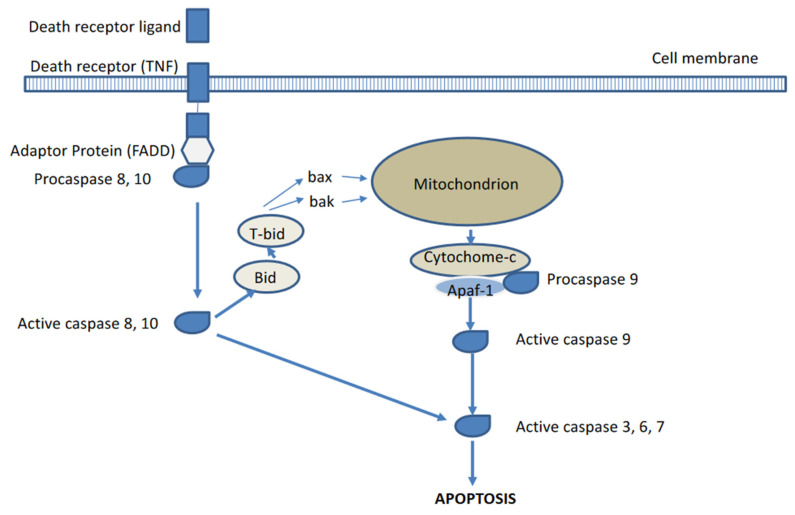
Extrinsic and intrinsic pathways of apoptosis activation.

**Figure 2:**
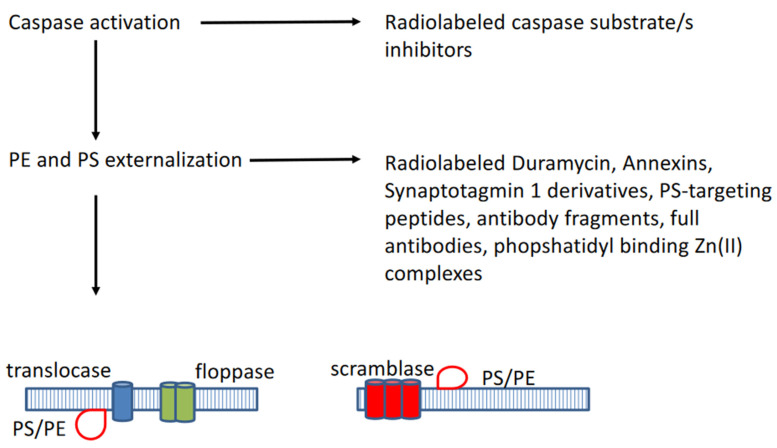
Following the activation of caspases, phosphatidylserine (PS) and phopshatidylethanolamine (PE) became externalized through activated enzymes such as translocase, floppase, and scramblase.

Two major pathways for apoptosis activation or induction have been described, respectively the extrinsic and intrinsic pathways (see Figure 1) [2,3,4]. The extrinsic pathway is activated via external cellular stimuli that result in the activation of cell membrane bound death receptors of the tumour necrosis factor (TNF) receptor superfamily such as CD95 (APA-1/Fas) or TNF-related apoptosis-inducing ligand (TRAIL) receptors by CD-95 ligand or TRAIL, which will result in receptor aggregation and recruitment of adaptor-molecule Fas-associated death domain (FADD) and pro-cysteine dependent aspartate-directed enzyme 8 (pro-caspase 8). FasR, FADD, and pro-caspase 8 together form the death-inducing signalling complex (DISC) where caspase 8 is activated. The activated caspase 8 will in turn activate the executioner pathway caspases (sequential conversion or activation of pro-caspase 3 to caspase 3, pro-caspase 6 to caspase 6, and pro-caspase 7 to caspase 7) to degrade cellular components. The intrinsic or mitochondrial pathway is initiated by internal cellular stress signals that will result in the release of mitochondrial cytochrome c into the cytosol which will then bind to an adaptor protein (APAF-1), which will recruit pro-caspase 9 resulting in the formation of a caspase activating multiprotein complex called the apoptosome. Once activated, caspase 9 will then also activate the executioner pathway caspases resulting in apoptosis. The extrinsic and intrinsic pathways are interconnected at several levels, for instance, caspase 8 may activate truncated-Bid (t-Bid), which may induce the cytochrome-c release by the mitochondria. Following the activation of the executioner caspase 3, morphological and biochemical cellular changes ensue (see Figure 2) such as externalization of phosphatidylserine (PS) and phosphatidylethanolamine (PE), which are normally confined to the inner membrane leaflet via a flip-floppase enzyme, acidification of the cell membrane, cytoplasm shrinkage and DNA degradation, cell shrinkage, cell membrane blebbing, and fragmentation of the cell into apoptotic bodies. The latter are subsequently removed by macrophages, in which the externalized PS constitutes an eat-me signal [2,3,4].

To date, a vast amount of preclinical data supports the notion that killing of tumour cells by the most currently available anticancer treatment agents used in routine clinical practice is linked to the rapid (within 48 h following treatment initiation) activation of apoptosis signal transduction pathways. In addition, failure to undergo apoptosis has been shown to result in treatment resistance. In the clinical setting, however, the occurrence of apoptosis following chemotherapy or radiotherapy is less well documented likely due to the need of sequential biopsies at appropriate times, which are currently unclear given the fact that different agents and treatment options induce apoptosis with different kinetics [2,3,4,5].

The current criteria used to define the response to cancer treatment are either based on morphological imaging that measure changes in tumour volume for solid tumours or on changes in tumour metabolism in lymphoma (interim FDG PET) occurring at the earliest 2 to 3 months following the effective treatment [6,7]. During this period, non-responders suffer from unnecessary treatment related toxic side effects and are at the same time deprived of a potential other beneficial treatment. Accordingly, imaging of apoptosis by means of whole body positron emission tomography, allowing for repetitive non-invasive in vivo detection of the site and the extent of apoptotic cell death obviating the need for invasive biopsy, is of major interest given the fact that it may potentially allow for a much more rapid assessment of treatment response to the cancer treatment (within 48 h following the treatment instigation), and thus, also avoid the subsequent administration of additional sessions of ineffective toxic treatment agents to non-responders. As opposed to single photon emission tomography (SPECT) imaging, positron emission tomography (PET) bears a higher sensitivity and allows for a more quantitative assessment of tracer uptake. To date, PET-imaging probes developed for apoptosis imaging include membrane-asymmetry targeting agents, cell membrane acidification targeting agents, and caspase substrates or inhibitors. In this paper, the available literature on these imaging agents in an oncological setting is reviewed.

## 2. Membrane-Asymmetry Targeting Agents

### 2.1. Phosphatidylethanolamine Targeting PET-Tracers

Duramycin, a 19-amino-acid antimicrobial peptide characterized by the presence of unusual thioether-linked amino acids generated through post-translational modifications and produced by streptomyces verticillium was shown to bind to phosphatidylethanolamine (PE), normally confined to the inner membrane leaflet but externalized during apoptosis, at a 1:1 ratio with relatively high affinity and exclusive specificity [8,9]. Binding seems to preferentially occur on highly-curved PE-rich membranes. Duramycin was radiolabelled with ^18^F by Yao et al., who found a 1.9-fold increase in the uptake of ^18^F-FP-duramycin by Jurkat cells following the anti-Fas treatment-induced apoptosis [10]. In contrast, after inhibition of the apoptotic process by the prior treatment with a caspase inhibitor (Z-VAD-FMK), the enhanced uptake of ^18^F-FP-duramycin was not detected. Of interest, the tracer uptake of necrotic cells was 1.4-fold higher than that of apoptotic cells. Furthermore, in S180 fibrosarcoma-bearing, A-549 human lung adenocarcinoma-bearing, and SPCA-1-bearing nude mice, dying cells in regions in tumours induced by CTX or cisplatine treatment were clearly visible on PET-images of mice injected with ^18^F-FP-duramycin when compared to normal organs and tumours of untreated mice. Li et al. further performed ^18^F-FB-duramycin and ^18^F-AI-NODA-duramycin uptake and blocking experiments using the leucocyte monocyte lymphoma cell line (U937) and flow cytometry as control [11]. The uptake of both tracers by U937 cells was enhanced in response to the degree of apoptosis and significantly inhibited by 70% and 61%, respectively for ^18^F-AI-duramycin and ^18^F-FB-duramyicin with cold duramycin. Moreover, in cells treated with 500 nM camptothecin (CPT), the uptake was specifically reduced by 14% and 12%, respectively. More recently, Rix et al. confirmed the specificity of ^68^Ga-NODAGA-duramycin for PE binding in vitro using competitive binding experiments, as well as in vivo in untreated doxorubicin, busulfan, and cisplatin-treated mice 2 h after intravenous injection of the ligand [12]. Organ toxicities were successfully determined using the ligand and confirmed by immunohistochemistry and blood parameter analysis.

### 2.2. Phosphatidylserine Targeting PET-Tracers

#### 2.2.1. Radiolabelled Annexins

Similar to phosphatidylethanolamine (PE), phosphatidyl serine (PS) is normally confined to the inner membrane leaflet but becomes externalized during apoptosis, and thus, constitutes an interesting target for apoptosis imaging. Various PS binding radiopharmaceuticals targeting PS have been developed, respectively radiolabelled Annexins, synaptotagmin I derivatives, PS-binding peptides, antibody fragments, and full antibodies, as well as phosphatidyl-Zn(II) complexes.

-^124^I radiolabelled Annexin V: Annexin V has been directly iodinated with ^124^I via the chloramine-T methods, as well as indirectly using pre-labelled N-succinimidyl-3-(^124^I)iodo-benzoate (^124^I-m-SIB-Annexin V) and (^124^)N-hydroxysuccynil-4-iodobenzoate (^124^I)4IB-Annexin V (12,13,14). The biological activity of ^124^I-iodinated Annexin V and ^124^I-m-SIB-Annexin V was tested in control and camptothecin-treated (i.e., apoptotic) human leukemic HL60 cells by Glaser et al. [13]. A significantly higher (21%) binding in the treated cells was observed with [^124^I]m-SIB-Annexin V. The binding of [^124^I]m-SIB labelled Annexin V to camptothecin treated cells was blocked (68%) by a 100-fold excess of unlabelled Annexin V. Inversely, in a study by Collingridge et al., ^124^I-m-SIB Annexin V failed to detect apoptosis in mice with 5-FU-treated RIF-1 tumours [14]. In addition, (^124^I)4IB-Annexin V was shown to bind to phosphatidylserine-coated microtiter plates and apoptotic Jurkat cells, as well as to accumulate in hepatic apoptotic lesions in mice treated with the anti-Fas antibody by Dekker et al. [15,16]. As compared to ^124^I-Annexin V, (^124^I)-4IB-Annexin V was shown to have a higher rate of binding to phosphatidylserine in vitro, a higher kidney and urine uptake, a lower thyroid and stomach content uptake, greater plasma stability, and a lower rate of plasma clearance. The same authors also synthesized ^124^I-maltose-binding protein-Annexin V (^124^I-MBP-Annexin V), which was shown to have a significantly higher binding to camptothecin-treated Jurkat cells when compared to the control cells and to exhibit a nine-time higher liver-uptake in mice treated with the anti-Fas antibody when compared to the untreated mice with histology, confirming the presence of apoptotic hepatocytes in anti-Fas-treated mice [17]. Unfortunately, a high thyroid tracer-accumulation was also identified indicative of rapid dehalogenation.

-^18^F-labelled Annexin V: ^18^F-labelling of Annexin V has been performed using the non-specific amine-directed conjugation group FSB (N-succinimyl-4-(18F)fluorobenzoate), which can conjugate to any of the 23 primary amines (22 lysine residues and one N-terminal amine) present on Annexin V, as well as using conjugation groups that specifically target the free thiol group of the single cysteine residue present on the wild-type Annexin V, respectively at position 315, such as N-(4-554-(^18^F)fluorobenzylidene)aminooxyl)butyl)maleimide((18F)FBABM) and (^18^F)FDG-maleimidehexyloxime (^18^F-FDG-MHO) [18].

Using in vitro experiments, ^18^F-FSB-Annexin V showed an intact binding to PS expressing red blood cells, a 60% increased binding to apoptotic Jurkat T-cell lymphoblasts (induced by irradiation) compared to non-apoptotic control cells, and an 88% higher binding to TC32 sarcoma-cells treated with the apoptosis-inducing agent etoposide compared to the untreated cells [19,20,21]. On pre-clinical PET imaging studies, the ligand showed the highest uptake in the kidneys and bladder, as well as a rapid clearance from the other organs in healthy Sprague-Dawley rats, a 3- to 9-fold increase in liver uptake in cycloheximide-induced, histologically confirmed, liver apoptosis in rats when compared to the control rats and a specific and time-dependent increase in uptake in UMC-SCC-22B tumour xenografts grown in mice, which were treated with two doses of doxorubicin with a 1-day interval. With regard to the latter study, the tumour uptake began to increase at the earliest imaging time point (6 h) and peaked at 3-days post-treatment, at a time when the tumour size was still unchanged.

Finally, in a calcium-titrated cell-binding study performed on red blood cells with exposed PS, both ^18^F-FBABM Annexin V and (^18^F)FDG-maleimidehexyloxime (18F-FDG-MHO) displayed a comparable binding to PS to that of wild-type Annexin V.

-^18^F-labelled Annexin B1: Annexin B1 is a novel member of the annexin superfamily that was isolated from a Cysticercus cellulosae cDNA library. Similar to Annexin V, it has been shown to bind to PS with high affinity. Wang et al. radiolabelled both Annexin V and Annexin B1 with ^18^F-FSB and performed PET/CT studies using both ligands 2 h post tracer injection in W256 tumour bearing rats treated with cyclophosphamide for 24 h [22]. The mean uptake ratios in tumours, confirmed to be apoptosis specific by means of haematoxylin staining and TUNEL-assays, as compared to that of contralateral muscle were 11.2 and 14.6, respectively for ^18^F-FSB-Annexin B1 and ^18^F-FSB-Annexin V.

-^64^Cu-labelled Annexin V: Cauchon et al. synthesized ^64^Cu-DOTA (1,4,7,10-tetraazacyclodecane-N,N′,N″,N‴-tetraacetic acid)-biotin, which was then exposed to an excess of streptavidin (SAv) to produce ^64^Cu-DOTA-biotin-SVa leaving three of the four biotin-binding sites available to bind to biotinylated Annexin V [23]. The authors first injected biotinylated Annexin V in tumour-bearing rats where tumour apoptosis was induced by photodynamic therapy (PDT) using phthalocyanine dyes as photosensitizers and red light, followed 2 h later by an avidine chase and yet another 2 h later by injection of ^64^Cu-DOTA-biotin-SAv. PET images were obtained up to 13 h post PDT delineated apoptosis in treated tumours and as early as 30 min following the ^64^Cu-DOTA-biotin-SAv injection, with tumour-to-background ratios reaching a maximum at 3 h post injection.

-^68^Ga-labelled Annexin V: Bauwens et al. radiolabelled the two Annexin V mutants, Cys2-Annexin V and Cys165-Annexin V, which contain a single cystein residue, respectively at the 2-position and the 165-position, with ^68^Ga-dota-maleimide [24]. The authors found that Jurkat cells treated with the anti-Fas treatment bound five times more ^68^Ga-Cys2-Annexin V and ^68^Ga-Cys165-Annexin V compared to the untreated cells. Furthermore, normal mice treated with anti-Fas showed a three to eight times greater hepatic tracer uptake compared to the untreated mice.

#### 2.2.2. Radiolabelled Synaptotagmin I Derivatives

Similar to Annexin V, the C2A domain of the 14.7 kDa protein synaptotagmin binds to PS in a calcium-dependent manner [25]. Heuting et al. synthesised a maleimide functionalised bis(thiosemicarbazone), H2ATSE/AMal, for site-specific ^64^Cu radiolabelling of thiol-functionalised C2Ac [26]. When radiolabelling was performed by incubation of the ligand-protein conjugate (post-labelling approach), analysis of the resultant ^64^Cu-ATSE/AMal-C2Ac revealed that the pre-labelled ^64^Cu-ATSE/AMal-C2Ac conjugate had a good stability in the serum and maintained target affinity in a red blood cell binding assay. Wang et al. synthesized ^18^F-C2A-GST (glutathion-S-transferase) by labelling C2A-GST with N-succinimidyl 4-(18)F-fluorobenzoate (^18^F-SFB) [27]. ^18^F-C2A-GST was found to specifically bind to apoptotic cells, whereas the biodistribution in mice showed that ^18^F-C2A-GST was mainly excreted from the kidneys and rapidly cleared from blood and nonspecific organs. The high focal uptake of ^18^F-C2A-GST in VX2 tumours grown in rabbits was identified after a single dose of paclitaxel, whereas no significant uptake prior to therapy was found in the tumour. However, the overall uptake values were low (SUV max of 0.47 treated versus 0.009 untreated). More recently, Bulat et al. reported on the synthesis of ^18^F-C2Am of N-5-(^18^F)fluoropentyl)maleimide [28]. Following the treatment of Colo205 and MDA-MB231 xenografted mice with the TRAILR2 agonist or 5-FU and doxorubicin, ^18^F-C2Am generated mean tumour-to-muscle ratios of 6.1 and 10.7 within 2 h of its administration. It was found that a 20% increase in CC3 (cleaved caspase 3) positivity generated a one unit SUV-increase in the post/pre-treatment increase.

#### 2.2.3. Radiolabelled Phosphatidyl-Serine Binding Peptides, Antibody-Fragments, and Antibodies

Using phage-display, Burtea et al. identified nine different Annexin V binding peptides [29]. Their alignment with amino acid sequences of relevant proteins revealed a frequent homology with Ca^2+^ channels, reminiscent of the function of annexins. IC50 values for competition with Annexin V for PS-binding were in the order of 10 to 15 nM for two of these peptides, respectively the hexapeptides PGDLSR and LIPKFF. Both PGDLSR and LIPKFF were successfully radiolabelled with (^18^F)FSB and (^18^F)FBAM using their N-terminal cysteine-containing equivalents CPGDLSR and CLIPKFF [30]. However, the radiolabelled version proved to be significantly less potent to interact with PS when compared to Annexin V (IC50 value in the low mM range versus nM range) and their in vivo stability proved to be very poor. LIPKFF was also labelled with ^18^F through the bioconjugation chemistry with FDG but the resulting radiolabelled peptide did not allow for adequate imaging of PS-expression on apoptotic tumour cells [31]. Ben Azzouna et al. radiolabelled PGDLSR with ^68^Ga via the conjugation of beta-alanine-NODAGA to the N-terminus [32]. However, this radiotracer also proved to be metabolically unstable in vivo, and thus, not useful for imaging.

A 14-amino acid synthetic peptide, FNFRLKAQKIRFG or PSBP-0, was derived from the corresponding region of the enzyme phosphatidylserine decarboxylase PSD (amino acids 351–364 of the enzyme from Chinese hamster ovary cells) known to bind effectively and specifically to PS, by Igarashi et al. [33]. A modified version of this peptide, the 14-mer synthetic PS-binding peptide FNFRLKAGAKIRGFG (PSBP-6), which was end-capped through conversion of the carboxylic acid to an amid, was labelled with ^64^Cu for PET imaging by Perreault et al. using an aminovaleric avid (AVa) linker and NOTA (1,4,7-triazacyclononane-triacetic acid), yielding ^64^Cu-NOTA-Ava-PSBP-6 in which IC50 values for PS-binding proved to be in the sub-millimolar range [34]. Following 60 min in mice plasma, 31% of the intact peptide could still be detected. In mice bearing EL4 tumour xenografts treated with cyclophosphamide and etoposide, a 1.3-time higher uptake was identified in treated versus untreated tumours 5 min following the tracer injection. However, the tracer was subsequently rapidly washed out and a difference between untreated and untreated tumours could no longer be discerned, likely due to proteolytic degradation and ligand binding to blood cells reducing its plasma availability for tumour uptake.

Stafford et al. radiolabelled Fab2 fragments derived from the human PS-binding antibody PGN635 with ^124^I [35]. Following the injection of this ligand in mice, no ^124^I labelled degradation products nor free ^124^I could be identified. Clear delineation of PC3 prostate carcinoma xenografts grown in mice was achieved by PET 48 h after injection (tracer uptake of 1.2%ID/g tissue, respectively). Radiation of the tumours with 15 Gy or the systemic treatment of the tumours with 10 mg/kg docetaxel increased tracer localization in the tumours.

Bavituximab, a phosphatidylserine-targeting antibody, was shown to destroy tumour blood vessels and to slow the growth of tumours in mice. Jennewein et al. labelled bavituximab with ^74^As and evaluated its use for the imaging of subcutaneous dunning prostate R3227-AT1 tumours in rats [36]. The tumour-to-liver ratio 72 h after injection was 22 for bavituximab compared with 1.5 for an isotype-matched control chimeric antibody of irrelevant specificity. Immunohistochemical studies showed that bavituximab was labelling the tumour vascular endothelium. Likewise, Kumar et al. were able to visualise LNCaP xenografts tumour grown in mice using ^64^Cu-labeled bavituximab by means of PET [34]. PGN635 is another monoclonal antibody that targets PS and that was radiolabelled with ^89^Zr for PET imaging [37]. The high accumulation of ⁸⁹Zr-PGN635 was observed in paclitaxel treated tumours undergoing apoptosis reaching 30%ID/g and tumour-to-blood ratios up to 13. The tumour uptake in control groups treated with vehicle or imaged with a non-binding antibody probe proved to be significantly lower.

#### 2.2.4. Radiolabelled Phosphatidyl-Binding Zn(II) Complexes

Zinc(II) macrocyclic coordination complexes with cyclic polyamine units as low-molecular-weight annexin mimics have a selective affinity for biomembrane surfaces enriched with PS, and are therefore useful for the detection of cell death [38]. The cell death binding specificity of ^11^C-CyclenZn2 was demonstrated by significantly different uptake rates in camptothecin-treated and control PC-3 cells in vitro [39]. PET imaging using ^11^C-CyclenZn2 showed that cyclophosphamide-treated mice exhibited a significant increase of uptake rate in the tumor at 60 min post injection, compared with the control mice. Another Zn-II containing macrocyclic coordination complex that was radiolabelled for apoptosis imaging is ^18^F-FB-DPAZn2 (4-^18^F-Fluoro-benzoyl-bis(zinc(II)-dipicolylamine). A significantly higher tumour uptake of this ligand was observed in adriamycin treated Hepa1-6 hepatocellular carcinoma-bearing mice when compared to the untreated tumours by Wang et al. [40].

## 3. Cell Membrane Acidification Targeting Radioligands

A group of small amphipathic molecules, termed the Aposense family, in which their cellular uptake is largely based on changes in cell membrane pH during apoptosis was designed by Damianovich et al. [41]. This group includes, amongst others, DCC (N,N′-didansyl-l-cystine), NST-732 (5-dimethylamino-1-naphtalene-sulfonyl-alpha-ethyl)fluoroalanine), and its derivatives DFNSH (dansylhydrazone) and DSNBA (4-(5-dimethylamino-naphthalene-1-sulfonamido)-3-(4-iodo-phenyl)butanoic acid), as well as ML-9 (butyl-2-methyl-malonic acid) and ML-10 (pentyl-2-methyl-malonic acid). The uptake of these molecules in apoptotic cells is assumed to be due to a reduction of the energy barrier of the cell membrane resulting from the scrambling process during early apoptosis accompanied by membrane acidification and (mono)-protonation of the aposense molecules, which are subsequently internalized by active scramblases (flip-flop mechanism) following which they bind to cytoplasmic proteins.

NST-732 has been radiolabelled with ^18^F and its uptake was shown to be increased in cell death induced in a lymphoma rodent model following irradiation [42].

While ^18^F-ML-10 was shown to accumulate in apoptotic MDA-MB-231 and MDA-MB-468 cells 72 h after treatment with paclitaxel in vitro, no significant increase of tracer accumulation was found in comparable murine xenografts following apoptosis induction (as demonstrated by cleaved caspase 3 levels) by paclitaxel [43]. Following the radiation treatment of human nasopharyngeal carcinoma xenografts (highly differentiated xenografts CNE1 and poorly differentiated xenografts CNE2), the tumour to muscle ratios were statistically different at both 24 and 48 h in CNE1 and CNE2 mice (ratios in CNE2 xeongrafts being higher than those in CNE1 xenografts) [44]. In another study on nude mice implanted with UM-SCC-22B tumours that were treated with two doses of doxorubicin by Demirci et al., the tumour to liver ratios of ^18^F-ML-10 proved to be significantly higher at days 3 and 7 post-treatment but not on day 1 [45]. The imaging potential of ^18^F-ML-10 was also assessed in human volunteers and patients [46]. In a series of eight healthy volunteers, ^18^F-ML-10 demonstrated a favourable dosimetry, biodistribution, stability, and safety profile, as well as binding to apoptotic cells. In subsequent phase I/II clinical trials in cancer patients, the potential of ^18^F-ML-10 for assessing the response to radiotherapy through apoptosis induction of primary and secondary brain tumours was assessed. Allen et al. studied 10 patients suffering from brain metastases that were treated with radiotherapy at 30 Gy in 10 daily fractions [47]. All patients underwent a ^18^F-ML-10 PET examination prior to therapy and after nine or 10 fractions of radiotherapy. All lesions studied were detected by ^18^F-ML-10 PET and a highly significant correlation was found between early changes on the ^18^F-ML-10 scans and later changes in tumour anatomical dimensions assessed using sequential MRI-imaging. Similar results were obtained by Sun et al. in a study of 29 patients, diagnosed with intracranial tumours that underwent a cyberknife treatment at 14-24 Gy in 1-3 fractions, as well as a baseline and 48 h post-treatment ^18^F-ML-10 PET scan [48]. It was found that malignant tumours tended to be more sensitive to the CK treatment but that the treatment outcome was not affected by the pre-CK apoptotic status of tumour cells, as assessed using ^18^F-ML-1P PET. Oborski et al. studied four glioblastoma multiforme (GBM) patients using a similar study setup to that of the previous studies. [49] In their study, the uptake changes of ^18^F-ML-10 over time by the GBM proved to be not clearly related to time-to-progression. The authors suggested that this may be due to the fact that the GBM patients were undergoing varying rates of cell death and growth.

## 4. Radiolabelled Caspase Inhibitors and Substrates

Given the different pathways for apoptosis that ultimately converge on the executive caspase 3, various types of caspase 3 inhibitors and substrates have been developed, many of which have been radiolabelled for PET imaging.

Radiolabelled Caspase 3 substrates: Su et al. developed ^18^F-CP18, a peptide based DEVD (caspase 3 substrate recognition motif) containing substrate-based compound, as a PET apoptosis imaging agent using click chemistry [50]. The ligand additionally contains a polyethylene glycol (PEG) chain and galactose moiety to facilitate transport across cell membranes. In mice, the radioligand was predominantly eliminated via the kidneys and urine and rapidly eliminated from blood. Dexamethasone-induced apoptosis of the thymus in mice was shown to result in a 6-fold increase of caspase activity and a 4-fold increase of ^18^F-CP18 retention. Using in vitro cell assays, Xia et al. were able to show the capase 3 dependent uptake of ^18^F-CP18 in U-87MG human glioblastoma cells when treated with 5-FU [51]. Furthermore, the in vivo microPET uptake of ^18^F-CP18 in corresponding tumour xenografts was shown to correlate with the ex vivo determined intra-tumour capase 3 activity, as well as to match the results obtained using TUNEL-assays and immunohistochemical staining for capase 3. Similar results were obtained by Rapic et al. in treated Colo205 cancer cells, which were treated with 5-fluorouracyl, irinotecan or a combination of both [52]. In humans, Doss et al. obtained whole body PET/CT scans at various time points, up to 170 min post ^18^F-CP18 injection in seven healthy volunteers, as well as blood and urine samples. ^18^F-CP18 was shown to be rapidly cleared via the kidneys with an average effective dose of 8.3 mSv for an injected activity of 555 MBq and a 1-h voiding interval [53]. Results from a phase II study (NCT01766622) assessing the potential of ^18^F-CP18 to visualise ongoing apoptosis in patients suffering from relapsed platinum resistant or refractory epithelial ovarian cancer treated with the SMAC mimetic drug birinapant are awaited (last update 4 December 2019). Engel et al. radiolabelled the *O*-benzylthreonine-containing substrate 2MP-TbD-AFC, a highly caspase 3 selective and cell permeable agent, with ^18^F-TBD [54]). The ligand was shown to accumulate in ovarian cancer cells (OVCAR-5 cells) in a caspase- and cisplatin-dependent fashion. Furthermore, the PET-imaging of Jo2-antibody induced liver apoptosis in mice showed a significant increase in ligand signal in the liver in treated versus untreated mice.

Hight al. reported on the potential of the peptide-based ^18^F-FB-VAD-fluormethylketone caspase 3 substrate to visualize apoptosis-induction in (V600E)BRAF colon cancer xenografts in mice using a combined BRAF-mutation inhibitor and a dual P13K/mTor inhibitor [55]. More recently, intracellular activatable PET-imaging probes targeting caspase 3 were reported by Chen et al. and Qiu et al. [55,56]. Chen et al. developed ^18^F-C-SNAT, which when activated by caspase 3 and glutathione reduction, undergoes intramolecular cyclization followed by self-assembly to form nano-aggregates in targeted cells that may thus enable the detection of apoptosis using PET [56]. Qiu et al. reported on the stimuli-responsive probe ^18^FDEVD-CYs(StBu)-PPG(CBT)-AmBF3 or (^18^F)I) [57]. Following the cellular uptake of (^18^F)I, the disulfide bond of cysteine can be cleaved in the reducing environment of the tumour cell and the peptide in (^18^F)I, which is activated following the drug-induced activation of capase3/7 through the cleavage of the DEVD sequence and release of the amino-group of cysteine. Subsequently, the 1,2-aminothiol group will undergo a bioorthogonal condensation with the 2-cyano group of CBT to form a macrocyclized product (1-dimer), and more hydrophobic oligomers will be self-assembled in situ due to intermolecular pi-pi stacking interactions, which will result in a high density of radioactive signal. (^18^F)I proved to be very stable in a phosphate-buffered saline and mouse serum. In addition, retention of the ligand in DOX-treated HeLa cells was 2.2-fold that in untreated cells.

Radiolabelled caspase 3 inhibitors: Various small molecular, 5-isatin-sulfonamide moiety containing, caspase 3 inhibitors have been radiolabelled for PET imaging. Nguyen et al. developed the subnanomolar affinity for caspase 3 inhibitor ^18^F-ICMT-11 [58]. This ligand was shown to bind to murine fibrosarcoma RIF-1 and human pulmonary LNM35 cells treated with etoposide and cisplatin in vitro (2-fold increase), as well as to 38C13 murine lymphoma xenografts in vivo treated with 4-hydroxy-cyclophosamide (4-HC) by up to 2-fold at 24 h post-treatment compared to the vehicle treatment. Furthermore, no uptake of the tracer was found by caspase 3 deficient human breast MCF-7 cancer cells following the treatment with 4-HC. Using dynamic PET-imaging, Glaser et al. found a 2-fold increase in the ^18^F-IMCT-11 uptake (versus baseline) by EL-4 mice xenografts 24 h following the treatment with cyclophosphamide and etoposide [59]. Similar results were reported by Witney et al. for the non-small cell lung cancer cells lines PC9 and A549 and EGFR PC9 low/mutant treated with cisplatin [60]. Inversely, in the carboplatin treated high mutant EGFR PC9 cell line where necrosis is the predominant mode of cell death, no change in the ^18^F-IMCT-11 uptake was found. In mice bearing 38C13 B-cell lymphoma, HCT116 colon tumours or MDA-MB-231 breast tumours, a different time-dependent increase in the ^18^F-IMCT-11 uptake was found between the 4-HC treatment (peak at 24 h) and birinapant treatment (peak at 4 h) by Nguyen et al. [61]. Finally, Challapalli et al. performed a biodistribution and dosimetry study with ^18^F-IMCT-11 in eight healthy volunteers and found the injection of the ligand to be well tolerated by all subjects with no serious adverse events being reported [62]. The mean average effective dose was 0.025 mSv/MBq.

Other radiolabelled isatin based inhibitors of caspase 3 have been reported for apoptosis imaging but with suboptimal in vitro and preclinical imaging results [63,64,65,66].

## 5. Discussion and Conclusions

Due to their potential for non-invasive, repetitive, and selective in vivo identification of the location and extent of baseline, as well as treatment-induced apoptotic cell death, the radiolabelled apoptosis-targeting tracers for PET-imaging are of major clinical relevance in oncology.

To date, a wide variety of potential PET-apoptosis imaging agents have been developed either targeting apoptosis-induced cell membrane asymmetry and acidification or caspase 3 activation (inhibitors as well as substrates).

The PE-binding antimicrobial peptide duramycin has been radiolabelled with ^18^F and ^68^Ga using different chelating agents. Additionally, their uptake by various tumour xenografts was shown to be enhanced following apoptosis-induction and chemotherapy, as well reduced following the administration of cold or unlabelled duramycin confirming its specificity. However, the biodistribution of ^18^F and ^68^Ga radiolabelled duramycin in mice demonstrated that these radiotracers are largely excreted via the hepatobiliary tract with a significant accumulation and retention in the liver and spleen, likely by reticuloendothelial cells, which will limit their usefulness for the assessment of a treatment response to chemotherapy for tumour lesions residing in both organs.

The reported phosphatidylserine targeting radiotracers include radiolabelled annexins, radiolabelled synaptotagmin I derivatives, radiolabelled PS binding peptides, PS targeting antibody fragments and antibodies, as well as radiolabelled phosphatidyl-binding Zn(II) complexes. With the exception of directly iodinated (^124^I) Annexin V, which proved to be unstable due to in vivo dehalogenation, all the other currently PET-radiolabelled Annexins, respectively ^124^I-m-SIB-Annexin V, ^124^I-4IB-Annexin V, ^18^F-FSB-Annexin V, ^18^F-FBABM-Annexin V, ^18^F-Annexin B1, ^64^Cu-DOTA-biotin-SVa-Annexin V, as well as ^68^Ga-Cys2-Annexin V and ^68^Ga-Cys165-Annexin V were shown to specifically bind to PS in vitro and their uptake was shown to be enhanced in the in vivo xenografted tumours in mice treated by means of chemotherapy, however, to a significantly variable degree. This may, in part, relate to the tumour model used, in which different tumour cell lines bear a different sensitivity to a similar chemotherapeutic agent, to differences in the chemotherapeutic concentration and exposure time, as well as to the different timing of imaging performed post-treatment. As shown previously by our group, the dynamics of apoptosis induction in a colorectal cancer model following a single-dose of different chemotherapeutics, respectively the VEGF-targeting bevacizumab, panitumumab targeting the epidermal growth factor receptor, 5-FU, irinotecan and oxaliplatin, differed significantly which was not surprising given the fact that all five chemotherapeutics exert their cytotoxic effects through different mechanisms [67]. Furthermore, given the fact that the microenvironment and blood supply between xenografts and human malignancies differ substantially, the results obtained for the timing of apoptosis imaging induction in the animals reported cannot be automatically extrapolated to humans. Thus, in the clinical setting, it will be imperative to initially characterize the time course of PET-radiolabelled annexins uptake by the treated tumours. Therefore, the apoptotic peak or peaks are accurately identified so that the early tumour response to therapy can be properly evaluated. While PET-radiolabelled synaptotagmin 1 derivatives were also shown to specifically target the ongoing apoptosis in treated tumour xenografts, their overall uptake proved to be low. Furthermore, for ^18^F-C2Am, a 20% increase in apoptosis positivity as assessed histologically generated only one SUV-increase in the post/pre-treatment increase, questioning its potential usefulness in the routine clinical practice. A number of Annexin V binding peptides have also been radiolabelled for PET-imaging, but these have proven metabolically unstable in vivo, and thus, not useful for imaging. Due to their small size, peptides usually exhibit rapid pharmacokinetics, and good tumour targeting characteristics, with the ability to penetrate into tumours efficiently. Thus, it may prove worthwhile to synthesize and radiolabel more stable variants of these peptides that are less susceptible to endo- and exopeptidases through the incorporation of D-amino acids, chemically modified amino-acids, and terminal end-capping in order to improve their stability, provided their biological activity is not altered thereby. Two PS-targeting antibodies, respectively Bavituximab and PGN635, have also been radiolabelled for apoptosis imaging by means of PET, respectively with ^74^As and ^64^Cu, as well as ^89^Zr. Of interest, ^74^As bavituximab was shown to specifically bind to PS over-expressed on nonapoptotic endothelial cells of tumour vessels. As shown by Ran et al., tumour blood vessels may indeed overexpress PS on their cell surface under certain conditions such as oxidative stress and in the presence of activating cytokines [68]. The preferential binding of PS targeting antibodies to tumoral endothelial cells, as opposed to cancer cells undergoing apoptosis, may, in part, also relate to their large molecular weight and the well documented elevated intra-tumoral interstitial pressure hampering the influx of macromolecules into the tumour tissue. The binding to the tumour vessel PS by this radioligand, as well as other PS-targeting radioligands should not pose a problem for the treatment response assessment, given the fact that baseline scans are to be compared to the post-treatment scans. Finally, comparable, favourable, and limited preclinical imaging data have been reported for the radiolabelled PS targeting phopshatidyl-binding Zn(II) complexes ^11^C-CyclenZn2 and ^18^F-FB-DPAZn2 when compared to the other aforementioned PS targeting radioligands.

The best validated cell membrane acidification and caspase 3/7 targeting radioligands are respectively ^18^F-ML-10 from the Aposense family and the radiolabelled caspase 3 substrate ^18^F-CP18. Following preclinical studies in xenografted tumour animal models with favourable results, both ligands were subsequently injected in healthy individuals and shown to bear favourable dosimetric and safety characteristics. However, in contrast to, for instance, ^99m^Tc-HYNIC-Annexin V, surprisingly, neither of both tracers was taken up by the bone marrow in the healthy individuals under study [69,70]. Removal of white and red blood cells from the bone marrow through apoptosis, primarily through engagement of the Fas death receptor following the homocellular or heterocellular Fas ligand production, plays a major role in the maintenance of hematopoietic cell homeostasis [71,72,73,74]. The major apoptotic population in normal bone marrow are immature erythroblasts. While an accurate estimate of the number of immature erythroblasts undergoing apoptosis is not feasible due to their unknown clearance rate, their number is likely substantial given the ineffective quote of the erythropoietic process described in healthy subjects. Thus, the clinical value of both radioligands as an apoptosis imaging agent in patients, for instance, as suggested by a small number of subsequent studies in patients suffering from primary or secondary brain malignancies using ^18^F-ML-10, remains to be proven and warrants further investigation.

## Data Availability

Not applicable.
